# Chest Port Placement in Freestanding Outpatient Vascular Access Centers

**DOI:** 10.7759/cureus.101586

**Published:** 2026-01-15

**Authors:** Richard J Gray, Sheetal Chaudhuri, Hao Han, John Larkin, Murat Sor, Gregg M Miller

**Affiliations:** 1 Interventional Radiology, Azura Vascular Care, Malvern, USA; 2 Clinical Data Analytics, Renal Research Institute, New York, USA; 3 Clinical Advanced Analytics, Fresenius Medical Care, Global Medical Office, Waltham, USA; 4 Strategic Operations, Renal Research Institute, New York, USA; 5 Interventional Radiology, Georgetown University, Washington, USA; 6 Interventional Nephrology, Azura Vascular Care, Malvern, USA

**Keywords:** chest ports, implanted chest port, implanted vascular access device, implanted vascular access port, long-term venous access device

## Abstract

Background

Ports have traditionally been inserted in hospitalized inpatients; however, there has been an increasing transition to outpatient placement by interventionalists in hospital imaging suites. To our knowledge, port implantation in nonhospital settings has not been reported in peer-reviewed literature. Here, we report our experience with port placement in freestanding outpatient vascular centers.

Methodology

The electronic medical record for 47 centers was retrospectively searched to identify port placements between January 1, 2012, and December 31, 2018. Data included indications, platelet inhibitor/anticoagulants, American Society of Anesthesiologists (ASA) classification, port type, site, tip position, peri-procedure medications, procedure time, and pain scores. Complications were determined by phone calls at 48-72 hours.

Results

No short-term malfunctions were reported. In total, 5,890 ports were placed for chemotherapy (n = 5,531), IV therapy (n = 77), antibiotics (n = 74), hyperalimentation (n = 19), phlebotomy (n = 7), medications (n = 4), miscellaneous (n = 74), and unknown (n = 104). Regarding ASA classifications, 1% (n = 65) were categorized as Class I, 20% (n = 1,203) as Class II, 78% (n = 4,592) as Class III, and 0.5% (n = 30) as Class IV. Overall, 3,712 were single-lumen power ports, 341 dual-lumen, 19 unknown, 7 arm, 1 other, and 1,810 were unspecified. There were 5,855 chest, 19 arm, 1 thigh, and 15 unspecified ports. Tips were positioned in the superior vena cava (n = 1,582), superior vena cava-right atrium (n = 497), right atrium (n = 272), inferior vena cava (n = 2), inferior vena cava-right atrium (n = 1), or not specified (n = 3,536). The mean procedure time was 29 minutes (range = 6-137). The mean peak pain score was 0.86 (range = 0-10). Complications (n = 33) included 16 emergency/hospital admissions <24 hours for port-site bleeding (2), infection (1), pneumothorax (1), EKG changes (1), respiratory symptoms (3), tachycardia (2), unconfirmed infection (1), fall (1), chest pain (1), syncope (1), pain (1), or other (1). Furthermore, 17 Other complications included unrelated/unconfirmed infection (4), death <30 days (1), shortness of breath (1), infection (1), reversal agent (1), hypoglycemia (1), fall (1), and other (7). No leaks were reported.

Conclusions

According to the study findings, port placement in outpatient centers appears to be safe and provides short-term effectiveness.

## Introduction

Until the late 1990s, ports were typically placed by surgeons in an operating room [1]; however, there has been an increasing transition to placement by interventionalists in hospital imaging suites [2-5]. A recent hospital study demonstrated a significantly lower infection rate after port placement for outpatients compared to placement for inpatients [6]. To our knowledge, port implantation in nonhospital settings has not been reported in peer-reviewed literature. Here, we report the short-term safety and effectiveness results for outpatient port implantation in dedicated freestanding vascular access centers.

## Materials and methods

The centralized electronic medical record (EMR) for 47 centers was retrospectively searched to identify patients who had undergone port placement between January 1, 2012, and December 31, 2018. Background data was gathered from the chart, with identifying information masked. Data collected included age, sex, race, indications for port placement, platelet inhibitor/anticoagulant use, American Society of Anesthesia (ASA) classification [7], Mallampati (MP) Class [8], port type, site, tip position, port priming with heparin/saline, contrast usage, peri-procedure medications including intra/post-procedure antibiotics and sedation, procedure time, fluoroscopy time, intra-procedure/post-procedure pain scores [9], and post-procedure length of stay, i.e., recovery to discharge. Short-term adverse events determined by routine phone calls within 48-72 hours after placement, and occasional sporadic phone calls thereafter, including, but not limited to, poor port function, hospital or emergency department (ED) admissions, infections, and bleeding, were tabulated from two separate quality assurance databases.

This retrospective review study was conducted under a protocol reviewed by Western Institutional Review Board (WIRB) (Puyallup, WA; WIRB#WO1-1004600-1). WIRB determined the study was exempt due to the use of existing patient data that was deidentified by the investigator, and informed consent was not required per Title 45 of the United States Code of Federal Regulations part 46.101(b)(4). The study was conducted in adherence with the Declaration of Helsinki.

Although techniques varied over time and among operators, the following were consistently practiced: All ports were placed by fellowship-trained interventional radiologists or interventional nephrologists. The insertion techniques used were in line with the most recent Society of Interventional Radiology (SIR) practice guidelines [10] and the recently published Japanese SIR guidelines [11]. Pre-procedure ultrasound evaluations were utilized to select an appropriate vein for port placement. Procedure suites were positive pressure and cleaned terminally between procedures commensurate with Occupational Safety and Health Administration (OSHA) [12], American Society for Health Care Engineering [13], and state Departments of Health regulations. Moderate sedation was administered at the discretion of the physician by a nurse or certified registered nurse anesthetist. Appropriate hand hygiene was performed, and the neck and chest were prepared and draped in accordance with the Centers for Disease Control and Prevention [14] and SIR guidelines [15]. Maximum barrier precautions, including sterile gowns, gloves, mask, hair covering, and aseptic ultrasound technique mandated by OSHA [12], were maintained.

Access into the target veins was accomplished under direct ultrasound guidance, and micro-access systems were utilized, followed by serial dilatation to place a peel-away sheath. The site on the chest wall or arm chosen for placement of the port reservoir was anesthetized, an incision was made, and blunt dissection was used to create a port pocket. The access catheter was tunneled from the port pocket site to the venotomy, and the ports were inserted into the subcutaneous pocket. Fluoroscopic guidance was used to assist port placement and evaluate the course of the port catheters to ensure a smooth catheter course without kinks. The venotomies were closed with subcuticular sutures and/or skin glue. The port incisions were closed in two layers with subcutaneous stitches to appose the dermis and a running subcuticular stitch with/without skin glue for the skin. The ports were primed per operator preference. A follow-up fluoroscopic X-ray was obtained to evaluate for pneumothorax and confirm the positions of the catheter tips.

## Results

No short-term malfunctions were reported. Patient demographics and clinical characteristics are presented in Table 1.

**Table 1:** Patient demographics and clinical characteristics. Patients’ physical status was determined by the American Society of Anesthesiologists (ASA) Physical Status Classification [7]. Airway status was determined using the Modified Mallampati (MP) Classification [8]. SAS Enterprise Guide Version 7.13. (SAS Institute Inc., Cary, NC, USA) was used to perform the data analysis [16].

Category	Subcategory	Number (n)	Percent (%)
Sex	Female	3,655	62
	Male	2,235	38
Age	Mean ± SD	62.9 ± 14 years	-
	Range	19–102	-
Race/Ethnicity	White	2,013	34.1
	Black	896	15.2
	Hispanic or Latino	593	10
	Multiracial	6	0.1
	Other	32	0.5
	Declined	1,182	20.4
	Not reported	1,168	19.8
ASA classification	I	65	1
	II	1,203	20
	III	4,592	78
	IV	30	0.5
MP classification	0	2,946	50
	I	527	8.9
	II	1,862	32
	III	528	9
	IV	27	0.5
Indications	Chemotherapy	5,531	91.4
	IV therapy	77	1.3
	Antibiotics	74	1.2
	Hyperalimentation	19	0.3
	Blood draws	7	0.1
	Medications	4	0.1
	Miscellaneous	74	1.2
	Unknown	104	1.7

There were 3,655 female and 2,235 male patients with a mean age of 62.9 ± 14 years (range = 19-102). Reported races were 2,013 White, 896 Black, 593 Hispanic or Latino, 6 multiracial, 32 others, 1,182 declined to specify, and 1,168 were not reported.

Overall, 5,890 ports were placed for chemotherapy (n = 5,531), long-term intravenous therapy (fluids/transfusions/pheresis) (n = 77), antibiotics (n = 74), hyperalimentation (n = 19), blood draws (n = 7), medications (n = 4), miscellaneous (n = 74), or unknown (n = 104). Overall, 15.4% (n = 906) of patients were on platelet inhibitors, and 5% (n = 301) were taking anticoagulants at the time of the procedure. Regarding ASA classifications, 1% (n = 65) were classified as Class I, 20% (n = 1,203) as Class II, 78% (n = 4,592) as Class III, and 0.5% (n = 30) as Class IV. MP classes were class 0 in 50% (n = 2,946), class 1 in 8.9% (n = 527), class 2 in 32% (n = 1,862), class 3 in 9% (n = 528), and class 4 in 0.5% (n = 27) of patients.

Port data is presented in Table 2. Overall, 3,712 were single-lumen power ports, 341 dual-lumen, 19 unknown, 1 other, and 1,810 were unspecified. There were 5,855 chest ports (4,901 right chest wall, 898 left chest wall, 56 chest wall laterality unspecified), 19 peripheral (12 right arm, 7 left arm), 1 thigh, and 15 unspecified port locations. When port tip position was reported, tips were positioned in the superior vena cava in 67% (n = 1,582), superior vena cava-right atrium in 21% (n = 497), right atrium in 12% (n = 272), inferior vena cava in 0.08% (n = 2), and inferior vena cava-right atrium in 0.04% (n = 1). Tip position was not specified in 60% (n = 3,536) of cases.

**Table 2:** Port data. SVC = superior vena cava; RA = right atrium; IVC = inferior vena cava

Category	Subcategory	Number (n)	Percent (%)
Port type	Single lumen	3,712	63.1
	Dual lumen	341	5.8
	Unknown	19	0.3
	Other	1	0.0
	Unspecified	1,810	30.8
Location	Chest (right)	4,901	83.2
	Chest (left)	898	15.2
	Chest (side unspecified)	56	1.0
	Peripheral (right arm)	12	0.2
	Peripheral (left arm)	7	0.1
	Thigh	1	0.0
	Unspecified	15	0.3
Tip position (specified)	SVC	1,582	67
	SVC-RA	497	21
	RA	272	12
	IVC	2	0.08
	IVC-RA	1	0.04
Tip position (not specified)		3,536	60

Table 3 presents the port procedure details. Overall, 13% (n = 765) of patients received a mean of 7.4 mL of contrast (range = 0-140 mL), at the operators’ discretion to facilitate negotiation of central vein tortuosity or stenosis. The other 87% (n = 5,125) of patients received no contrast (n = 4,876) or an unknown quantity because the amount given was not in a searchable field (n = 249).

**Table 3:** Port procedures. Pain scores were obtained using Wong-Baker FACES® Pain Rating Scale [9].

Category	Subcategory	Number (n)	Percent (%)
Contrast utilization	Used	765	13
	None or unknown dose	5,125	87
Medications	Antibiotics	3,896	66
	Fentanyl	4,692	80
	Midazolam	3,846	65
	Propofol	1,137	19
	Oxycodone	495	8.5
	Oxycodone/Acetaminophen	256	4.3
Locking solution	Heparin/Heparinized saline	5,165	87.7
	Saline	650	11
	Unknown	75	1.3
Procedure time	-	Mean ± SD = 29 ± 13 minutes	-
Pain scores	Intraoperative	Mean = 0.53	-
		Range = 0–10	-
	Postoperative	Mean ± SD = 0.1 ± 0.78	-
		Range = 0–10	-
Post-procedure length of stay	-	Mean ± SD = 33.1 ± 16.0 minutes	-

Overall, 66% (3,896) of patients received antibiotics either pre-procedure 8.9% (n = 523), intra-procedure 53% (n = 3,106), or post-procedure 4.5% (n = 267). Moreover, 80% (n = 4,692) of patients received fentanyl, 65% (n = 3,846) received midazolam, 19% (n = 1,137) received propofol, 8.5% (n = 495) received oxycodone, and 4.3% (n = 256) received oxycodone/acetaminophen. Ports were locked with heparin/heparinized saline in 87.7% (5165), saline in 11% (650), or unknown in 1.3% (75) of patients.

The mean procedure time was 29 ± 13 minutes. The maximum intraoperative pain score was a mean of 0.53 (range = 0-10). The maximum postoperative pain score was a mean of 0.1 ± 0.78 (range = 0-10). The mean post-procedure length of stay was 33.1 ± 16.0 minutes. 

Complications are summarized in Table 4. Complications occurred in 0.56% (n = 33) of patients. These included 16 ED or hospital admissions within 24 hours for port-site bleeding (2), port infection (1), pneumothorax (1), EKG changes (1), shortness of breath (2) respiratory failure unresponsive to reversal agents (1), tachycardia (2), signs or symptoms of infection unconfirmed by subsequent workup (1), fall at home (1), chest pain (1), syncope (1), pain (1), or other (1). Moreover, 17 other reported complications included signs or symptoms of infection unrelated or unconfirmed by subsequent workup (5), death <30 days (1), shortness of breath (1), reversal agent used (1), symptomatic hypoglycemia (1), fall without injury (1), and other (7). There were no cases of hemothorax or cardiac tamponade. No leaks were reported.

**Table 4:** Complications.

Category	Subcategory	Number (n)
Port-related complications (hospital admission <24 hours)	Port site bleeding	2
	Pneumothorax	1
	Port Infection	1
	Port malfunction	0
	Hemothorax	0
	Cardiac tamponade	0
	Leaks	0
Procedure-related emergency department or admissions (<24 hours)	EKG changes	1
	Shortness of breath	2
	Respiratory failure (unresponsive to reversal agents)	1
	Tachycardia	2
	Unconfirmed infection	1
	Fall at home	1
	Chest pain	1
	Syncope	1
	Pain	1
	Other	1
Other complications	Infection (unrelated/unconfirmed)	5
	Death <30 days	1
	Shortness of breath	1
	Reversal agent used	1
	Symptomatic hypoglycemia	1
	Fall without injury	1
	Other	7

## Discussion

There were no short-term malfunctions or port leaks after placement in our nonhospital outpatient vascular access center suites. Therefore, our immediate success rate is much higher than previously reported literature success rates of 90% [10] and well above the SIR quality improvement guideline threshold of 95% [10]. All procedures were performed using ultrasound and fluoroscopic guidance. Similarly, port placement in hospital interventional suites using image guidance is performed with high success and low complication rates [3-5,17]. Ultrasound guidance significantly decreases catheter placement failure [18,19], complications [18,19], including pneumothorax [19], arterial puncture [19], and multiple catheter placement attempts [18,19] compared with blind or external landmark placement techniques [10,18,19]. The SIR quality improvement guidelines [10] concur that the incidence of early complications using ultrasound and fluoroscopy is lower compared to blind or external landmark techniques.

Most (78%) of our patients were categorized as ASA Class III, i.e., they had severe systemic disease with substantive functional limitations [7]. Despite this sick patient population, the procedures were short (mean <30 minutes), required minimal fluoroscopy (mean <1 minute fluoroscopy time), were well tolerated under moderate sedation with a mean maximum pain score <1 (scale 1-10), and required post-procedure recovery times <40 minutes. Even with this sick population, we encountered a very low short-term complication rate of <0.6%, which is well below the SIR quality improvement guideline thresholds [10] for immediately apparent complications, including pneumothorax (4%), hemothorax (2%), hematoma (4%), perforation (2%), and air embolism (2%). We found no port leaks and a tiny fraction of direct port complications, including pneumothorax, port-site bleeding, and infection. Our complication rate would be anticipated to be higher with longer-term follow-up, for which the reported literature rate of major and minor complications is approximately 7% of patients [3,4,10,20].

Our patients’ needs for a port were typical. In our study, the predominant indication for port placement was long-term venous access for medication administration, predominantly chemotherapy, which is similar to previous reports [1,20-22]. As in our study, central venous ports are also placed in patients who need fluid administration [2], contrast administration [2,21], repeated blood draws [2,21,22], plasmapheresis, parenteral nutrition, and blood transfusions [10,22].

Two-thirds of our patients over the seven-year period of our review received prophylactic antibiotics out of concern for early infection and to comply with local hospital practices, while the rest received no antibiotic prophylaxis. This inconsistency reflects the lack of consensus in medical literature. Some studies show a lower complication rate when antibiotics are given [23], while other studies [21,24-26] suggest no advantage with strict aseptic care before and after port placement [21]. The most recent SIR guidelines [27] for antibiotic prophylaxis considered the benefit of antibiotics for port placement to be unproven, their routine use controversial, and therefore failed to reach a consensus, citing lack of evidence. On the other hand, the Japanese SIR, citing allergic reaction potential and increased microbial resistance, recently recommended that prophylactic antibiotic administration be limited to at-risk patients, for instance, immunocompromised patients [11].

When known, a majority (67%) of the catheter tips in our study were positioned in the superior vena cava, near the right atrial junction, as determined by fluoroscopy. This is consistent with the Japanese SIR guidelines that recommend tip placement in the superior vena cava using fluoroscopic measurements [11]. Furthermore, the most recent SIR [10] guidelines also recommend fluoroscopy to determine the port catheter tip position. Studies have varied regarding optimal tip position, with some studies suggesting better function with the tip in the right atrium [28]. while others have reported cardiac tamponade [29]. We had no incidents of cardiac tamponade in the 272 patients with the catheter tips positioned in the right atrium.

According to the Centers for Medicare and Medicaid Services Physician Supplier Procedure Summary File [30] of Medicare beneficiaries, in 2022, only 9.7% of chest port insertions were performed in nonhospital-affiliated ambulatory surgery centers or physician offices. Most chest ports were placed in a hospital setting for outpatients (77%) or for inpatients (10.8%). Our study is, to our knowledge, the first report of port insertions in freestanding nonhospital outpatient centers and the largest report of port implantations in the literature for any setting, dwarfing other reports. Our study is limited by its retrospective nature, incomplete reporting of some data points, lack of a comparison control group, and short-term follow-up. Nevertheless, we describe no short-term malfunctions and very few complications. The insertion of ports was performed by many operators at many centers, which reflects the real-world experience of board-certified interventional radiologists and interventional nephrologists in freestanding access centers.

## Conclusions

The short-term safety and effectiveness results for outpatient port implantation performed by fellowship-trained interventional radiologists and nephrologists revealed no short-term malfunctions and a tiny direct port complication rate well below prior reports. The insertion of ports by many operators at many centers reflects the real-world experience for outpatient port implantation in dedicated freestanding vascular access centers. Therefore, we conclude that port placement in freestanding outpatient centers by appropriately trained practitioners appears to be safe and suitable to provide short-term effectiveness.
